# Low-dose oncolytic adenovirus therapy overcomes tumor-induced immune suppression and sensitizes intracranial gliomas to anti-PD-1 therapy

**DOI:** 10.1093/noajnl/vdaa011

**Published:** 2020-02-03

**Authors:** Zineb Belcaid, Cor Berrevoets, John Choi, Edward van Beelen, Eftychia Stavrakaki, Tessa Pierson, Jenneke Kloezeman, Denis Routkevitch, Mariëlle van der Kaaij, Alicia van der Ploeg, Dimitrios Mathios, Stefan Sleijfer, Clemens Dirven, Michael Lim, Reno Debets, Martine L M Lamfers

**Affiliations:** 1 Department of Neurosurgery, Brain Tumor Center, Erasmus University Medical Center, Rotterdam, The Netherlands; 2 Laboratory of Tumor Immunology, Department of Medical Oncology, Erasmus MC Cancer Institute, Rotterdam, The Netherlands; 3 Department of Neurosurgery, Johns Hopkins Hospital, Johns Hopkins University School of Medicine, Baltimore, Maryland, USA

**Keywords:** Delta24-RGD, glioma, immunotherapy, oncolytic virotherapy, PD-1

## Abstract

**Background:**

The tumor-selective human adenovirus Delta24-RGD is currently under investigation in phase II clinical trials for patients with recurrent glioblastoma (GBM). To improve treatments for patients with GBM, we explored the potential of combining Delta24-RGD with antibodies targeting immune checkpoints.

**Methods:**

C57BL/6 mice were intracranially injected with GL261 cells and treated with a low dose of Delta24-RGD virus. The expression dynamics of 10 co-signaling molecules known to affect immune activity was assessed in tumor-infiltrating immune cells by flow cytometry after viral injection. The antitumor activity was measured by tumor cell killing and IFNγ production in co-cultures. Efficacy of the combination viro-immunotherapy was tested in vitro and in the GL261 and CT2A orthotopic mouse GBM models. Patient-derived GBM cell cultures were treated with Delta24-RGD to assess changes in PD-L1 expression induced by virus infection.

**Results:**

Delta24-RGD therapy increased intratumoral CD8^+^ T cells expressing Inducible T-cell co-stimulator (ICOS) and PD-1. Functionality assays confirmed a significant positive correlation between tumor cell lysis and IFNγ production in ex vivo cultures (Spearman *r* = 0.9524; *P* < .01). Co-cultures significantly increased IFNγ production upon treatment with PD-1 blocking antibodies. In vivo, combination therapy with low-dose Delta24-RGD and anti-PD-1 antibodies significantly improved outcome compared to single-agent therapy in both syngeneic mouse glioma models and increased PD-1^+^ tumor-infiltrating CD8^+^ T cells. Delta24-RGD infection induced tumor-specific changes in PD-L1 expression in primary GBM cell cultures.

**Conclusions:**

This study demonstrates the potential of using low-dose Delta24-RGD therapy to sensitize glioma for combination with anti-PD-1 antibody therapy.

**Key Points**

- Oncolytic virotherapy with Delta24-RGD synergizes with anti-PD-1 therapy.
- Delta24-RGD oncolytic virus therapy overcomes glioma-induced immune suppression.

**Importance of the Study**

Current subdued response rates in anti-PD-1 therapy in GBM patients emphasize the need to identify sensible strategies to overcome tumor-induced immune suppression. Oncolytic viruses are excellent candidates for combination therapy with immune checkpoint inhibitors due to the tumor-specific cell killing and activation of the adaptive immune system through immunogenic cell death. In this study, we selected a combination viro-immunotherapy strategy after meticulous assessment of co-signaling molecules expression in GBM tumors. We demonstrate that a low dose of the oncolytic adenovirus Delta24-RGD significantly alters the immune microenvironment in murine glioma, in particular by upregulation of PD-1 expression on CD8^+^ T cells. Combined Delta24-RGD therapy and PD-1 blockade resulted in increased overall survival in both the GL261 and CT2A glioma model. This study is the first to show that a low dose of Delta24-RGD is already sufficient to prime the immune system for effective synergy with anti-PD-1 antibody therapy.

Glioblastoma (GBM) is the most common and aggressive primary brain tumor in adults, with a median overall survival of 14.6 months for standard of care chemoradiotherapy^1^and 15.7–20.9 months in randomized clinical trials testing bevacizumab^2,3^ or tumor-treating fields.^4^ Despite intensive efforts over the past decades to develop new therapies for this tumor, none have significantly impacted patient mortality. The past years, expectations have arisen that the path to improved prognosis for GBM patients lies within the emerging field of immunotherapy. Immunotherapy with immune checkpoint inhibitors has rapidly gained ground and is a proven effective therapy for patients with certain forms of cancer.^5–9^ Immune co-signaling receptors determine the stimulation and inhibition of the T-cell response^10^ and tumor cells are able to exploit these immune signaling pathways to evade detection and clearance by the adaptive immune system. Targeting CTLA-4 and PD-1 has been proven successful to counteract this immunosuppressive mechanism.^6^ Human anti-PD-1 antibodies, Nivolumab and Pembrolizumab, inhibit the interaction between the PD-1 receptor and its ligands, PD-L1 and PD-L2, and are registered for the treatment of advanced melanoma, non-small cell lung carcinoma, bladder cancer, renal cell cancer, and tumors with mismatch repair deficiencies, irrespective of the exact tumor type. In recent clinical trials for GBM, however, monotherapy with Nivolumab or Pembrolizumab,^11,12^ as well as the combination with radiation therapy (NCT02667587), did not significantly improve survival.

Increased efforts are therefore currently ongoing to enhance antitumor activity from immunotherapy.^13^ One important focus includes the mechanism of the PD-1/PD-L1 axis mediated immune resistance leading to loss of T-cell effector functions. PD-L1 can be constitutively expressed on the surface of GBM cells through oncogenic signaling^14^ or alternatively expressed in response to immune-stimulating cytokines such as IFNγ, a mechanism called adaptive immune resistance.^15^ Topalian et al.^7^ showed that anti-PD-1 therapies are most likely unsuccessful in tumors without PD-L1 expression. PD-L1 expression has been correlated with histological subtypes in GBM, in particular with the Isocitrate dehydrogenase 1 (IDH1) status.^16^ Various other mechanisms in the GBM microenvironment contribute to the tumor-induced immune suppression,^17^ suggesting that effective antitumor therapy requires targeting from multiple angles. Indeed, antibodies administered as single agents against the checkpoints PD-1, CTLA-4, CD137 (4-1BB), or TIM-3 have been proven less effective than combination therapies against mouse intracranial GL261 tumors.^18–20^ These antibodies synergize effectively when combined with radiation therapy,^18–20^ chemotherapy,^21^ or vaccines.^22^ The rationale is that these latter therapies may induce immunogenic cell death to release tumor antigens into the tumor microenvironment, enhance major histocompatibility complex (MHC) molecule expression to increase antigen presentation, upregulate pro-inflammatory cytokines and chemokines, and increase trafficking of T cells into the tumor and activation of intratumoral T cells. We hypothesize that oncolytic virus (OV) therapy represents a promising approach for combination therapy with immune checkpoint inhibitors. Indeed, the tumor-specific cell killing and the activation of the adaptive immunity through immunogenic cell death to induce antigen-specific memory are important mechanisms conferred by OV therapy.^23^ One other important advantage of OV therapy over standard radiation therapy and chemotherapeutic agents is the selective killing of tumor cells without causing systemic immune suppression.^24,25^

In our studies, we applied the human adenovirus Delta24-RGD, also known as DNX-2401, which is genetically modified to selectively replicate in cells with a defective Retinoblastoma pathway causing cell death and release of viral progeny to infect neighboring tumor cells.^26,27^ We and others previously reported that this virus significantly improves survival in mouse models of malignant glioma, leading to 14–50% long-term survivors.^27–32^ We also showed that an intact adaptive immune system is fundamental in the OV-mediated induction of a long-lasting protective antitumor immune memory.^28,29^ Additionally, a phase I study in which patients with recurrent GBM were treated with Delta24-RGD reported that tumor specimens collected after intracranial virus therapy contained higher CD4^+^ and CD8^+^ T-cell infiltrates compared to pretreatment specimens.^33^ In the present study, we first determined which T-cell co-signaling receptor to target in combination with Delta24-RGD therapy, prior to treating mouse gliomas with a combination of Delta24-RGD with an antibody against the selected T-cell co-signaling receptor. As treatment of GBM with both replicating as well as non-replicating adenoviruses has been associated with self-limiting local inflammatory responses leading to edema-related symptoms like nausea and headache,^33–36^ we chose to test a single low dose of virus with the aim of inducing a degree of immunogenic cell death while limiting the risk of potential toxicity due to excessive inflammatory responses in the intracranial setting. We demonstrate that low-dose Delta24-RGD significantly upregulates PD-1^+^ expression by intratumoral CD8^+^ T cells and synergizes with PD-1 blockade to induce long-term survival. Our data further support the rationale to combine Delta24-RGD therapy with anti-PD-1 antibody therapy for patients with GBM. This combination strategy is currently under investigation in phase II clinical trial (NCT02798406).

## Materials and Methods

### Cells and Reagents

The murine GL261 glioma tumor was purchased from the NCI Tumor Repository, the murine CT2A glioma tumor cell line was kindly provided by Dr. Peter Fecci (Duke University), and the human lung carcinoma cell line A549 were purchased from the American Type Culture Collection. Cell lines were cultured in DMEM supplemented with 10% fetal bovine serum (FBS) and 1% penicillin–streptomycin. Patient-derived GBM cell cultures were established and cultured under serum-free conditions as previously described^37^ and maintained in serum-free culture medium containing DMEM-F12, 1% penicillin–streptomycin, 2% B27 (Invitrogen), human EGF (20 ng/mL), human bFGF (20 ng/mL) (both Tebu-Bio), and 5 μg/mL heparin (Sigma-Aldrich). All samples were obtained with informed consent from the patients and with approval of the medical ethics committee of the Erasmus Medical Center. Hamster anti-PD-1 monoclonal antibodies (clone G4) were produced as previously described.^38^ Hamster IgG control antibody was purchased from Bioxcell. Hamster anti-ICOS activating antibody (clone C398.4A) was purchased from BioLegend.

### Delta24-RGD

The construction of human Ad-Delta24-RGD virus has been described by Suzuki et al.^26^ The viral stocks were produced as previously described.^32^ In short, the virus was purified from infected cells and supernatant using the AdEasy Virus Purification Kit (Stratagene). The virus titer of the produced batch was assessed in A549 cells using the Adeno-X Rapid Titer Kit (Clontech).

### In Vivo Treatment Protocol

Six- to eight-week-old female C57BL/6J mice (Harlan) were maintained in individually ventilated cages. Animal procedures were performed in accordance with the local guidelines and approved by the Animal Ethics Committee at Erasmus Medical Center (protocol EMC3221, 125-14-01) and by the Animal Care and Use Committee at the Johns Hopkins University (protocol MO18M301). The immune-competent intracranial GL261 and CT2A glioma models were established as previously described.^28,39^ In short, mice were stereotactically injected with 5 × 10^4^ GL261 or CT2A cells in the right hemisphere with the following coordinates: 3 mm deep, 2.2 mm lateral, and 0.5 mm anterior of bregma. Mice were randomly assigned to treatment groups. On day 5 after tumor implantation, using the same burr hole, mice were treated by one intracranial injection with 2 × 10^7^ pfu Delta24-RGD in 10 µL PBS. This dose is approximately 10-fold lower than the dose used in previous therapeutic studies in the GL261 mouse model.^28–31^ In the immune cell kinetic studies, mice were sacrificed after 24 h, 48 h, 72 h, 7 days, 9 days, and 14 days by cervical dislocation under isoflurane anesthetic. Additional mice (*n* = 8) were treated following the same protocol and were euthanized on day 14 to assess PD-1 and ICOS expression and establish ex vivo brain tumor cell cultures. In the survival studies, mice were treated with 2 × 10^7^ pfu Delta24-RGD in 10 µL PBS followed by 3 cycles of 200 µg of anti-PD-1 or hamster IgG control antibodies per intraperitoneal injections on days 7, 9, and 11. Mice were monitored 3 times a week for weight loss, neurological symptoms, or signs of lethargy and were euthanized by cervical dislocation under isoflurane anesthetic upon indication or at the end of the experiment at 100 days post-tumor implantation.

### Processing and Flow Cytometry Staining of Brain Tumor-Infiltrating Lymphocytes and Splenocytes

Brains and spleens were collected from 3 to 5 mice from the PBS control and virus treatment groups on 24 h, 48 h, 72 h, 7 days, 9 days, and 14 days after Delta24-RGD therapy. Single cells from brain tumors were filtered through a 70 µm cell strainer, centrifuged at 1400 rpm, and resuspended in 4 mL of 60% Percoll (GE Healthcare Life Sciences). The cells were overlaid onto a 30%/37%/60% Percoll gradient and immune cells were collected from the 37%/60% interphase. Matched splenocytes were filtered through a 40 µm cell strainer, underlaid with 5 mL of Ficoll, and immune cells were collected from the interphase. Immune cells were stained with relevant antibodies for flow cytometry (see Supplementary Table S1) and analyzed on a FACSCanto flow cytometer and data analysis was performed with FCS Express 4 Flow Research software. Gating of co-signaling expression of interest within tumor-infiltrating lymphocytes (TILs) and splenocytes was performed as follows: (1) removal of doublets within the FSC H/W gates, (2) selection of lymphocytes within the FSC/SSC gates, (3) T-cell selection within the SSC/CD3 gates, (4) selection of CD4^+^ and CD8^+^ T cells, and (5) positive co-signaling expression selected through gating using Fluorescence Minus One controls.

### Ex Vivo Mouse Brain Tumor Cell Cultures

Brains were collected at 14 days after virus therapy and brain tumor dimensions were estimated by measuring the width, length, and height using a caliper. The tumor-bearing hemispheres were carefully dissected from the brain and cross-cut into 2 halves. The length and height of the tumor were measured and followed by another cross-cut perpendicular to the first cut to measure the width of the tumor. Tumor volumes were calculated based on the formula to calculate spherical volumes: 4/3π × radius (width) × radius (length) × radius (height). After dissociation, 10% of the entire suspension was cultured in complete T-cell medium (RPMI 1640 medium supplemented with 10% FBS, 1% penicillin-streptomycin, 25 mM HEPES, 200 nM l-Glutamine, 1% MEM non-essential amino acids, 1 mM sodium pyruvate, 50 µM β-mercaptoethanol, and 50 IU/mL rIL-2) in 25 000 cells/well in a 24-wells plate and incubated in the IncuCyte for live monitoring (Essen Bioscience). After 5 days of culture, loss of tumor cells was calculated by comparing images from the start to the end of culture with ImageJ software. The supernatant was collected and the production of IFNγ was assessed by ELISA (eBioscience).

### Splenocytes and GL261 Co-cultures

Spleens were harvested on day 14 after Delta24-RGD therapy and mechanically dissociated using a 40 µm cell strainer and cultured for 2 days in a complete T-cell medium. One day prior to co-culture, 5000 GL261 cells per well were seeded in a 24-wells plate. The following day, 10^6^ splenocytes and 5 µg/mL monoclonal antibodies (anti-PD-1, anti-ICOS, IgG) were added to the GL261 cells. The co-cultures were incubated for 4 days after which supernatant was collected to assess IFNγ production by ELISA (eBioscience).

### PD-L1 Expression in GL261 and Primary GBM Cell Cultures

For PD-L1 analysis, 10^5^ GL261 cells were plated in 24-wells plate and infected with Delta24-RGD 24 h later with the following multiplicities of infection (MOI): 10, 100, and 1000. After 24 h, cells were harvested and stained with 7-AAD (BD Biosciences) and anti-mouse CD274 (clone MIH5, eBioscience) and analyzed with flow cytometry. For PD-L1 analysis, 10^5^ cells from each of 12 low-passage patient-derived GBM cultures were plated in ECM-coated T75 flasks and allowed to adhere for 24 h. Media were supplemented with 10 ng/mL IFNγ or with a Delta24-RGD dilution corresponding to the MOI for each cell culture-specific IC_50_ value. After 24 h, cells were harvested and stained with 7-AAD (BD Biosciences) and anti-human CD274 (clone MIH1, eBioscience) and analyzed with flow cytometry.

### Immunohistochemistry

Sections (10 µm) from snap-frozen mouse brains fixed with acetone were blocked for 60 min (DAKO REAL Peroxidase-blocking solution, DAKO serum-free protein block) and incubated for 60 min with antibodies against adenovirus (Millipore), CD8α (clone 53–6.7, eBioscience), or CD4 (clone GK1.5, eBioscience) followed by the secondary antibodies rabbit anti-goat IgG or goat anti-rat IgG (Invitrogen). Sections were stained with DAB chromogen (DAKO) and hematoxylin (Sigma-Aldrich) and mounted with Pertex (Histolab). Sections were photographed on an Axio Observer D1 microscope with 40× magnification (Zeiss).

### Statistical Analysis

Comparison of immune subsets between virus and control groups was performed with the Student’s *t*-test. Correlation analyses were performed by calculation of the Spearman’s *R*. Survival was analyzed with the log-rank Mantel–Cox test. Statistical analyses were performed using GraphPad Prism 5 (GraphPad Software Inc.).

## Results

### Delta24-RGD Therapy Significantly Increases Local ICOS and PD-1 Expression in Brain TILs and Not in Splenocytes

As local intratumoral delivery of Delta24-RGD induces antigen-specific splenocytes in glioma-bearing mice,^28^ we hypothesized that treatment with Delta24-RGD induces changes in expression of co-signaling molecules on local and systemic T cells. To test this, we harvested brains and spleens from tumor-bearing mice at different time-points after Delta24-RGD therapy and assessed T-cell expression levels of 10 selected co-signaling molecules by flow cytometry. Overall, the relative expression of the co-signaling molecules in the splenocytes did not yield major differences between virus-treated and untreated mice. In general, the expression of co-signaling molecules was low (<20%) with the exception of CD28, ICOS, and PD-1which all showed a slight decrease over time (Supplementary Figure S1).

**Fig. 1:**
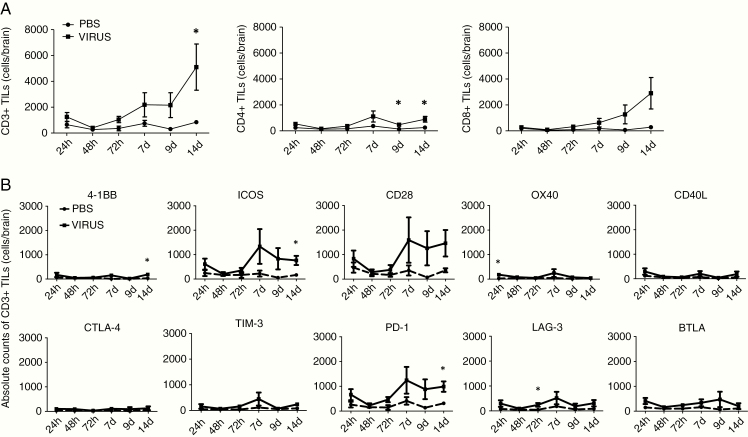
Delta24-RGD therapy significantly increases numbers of intratumoral ICOS^+^ and PD-1^+^ CD3^+^ T cells. C57BL/6J mice were inoculated i.c. with 5 × 10^4^ GL261 cells and after 5 days treated i.c. with 2 × 10^7^ pfu Delta24-RGD and control mice were treated with 10 µL PBS. Mice were sacrificed after 24 h, 48 h, 72 h, 7 days, 9 days, and 14 days post-therapy and cells were processed for flow cytometry (*n* = 3–5 mice/time-point/group). (A) Delta24-RGD significantly increases intratumoral CD3^+^ T cells on day 14 after therapy, reflecting an increase in CD8^+^ T cells. (B) Absolute numbers of CD3^+^ T cells were assessed and the expression of each co-signaling receptor within CD3^+^ T cells is analyzed. On day 14 after Delta24-RGD therapy, there was an increase in ICOS^+^ and PD-1^+^ TILs. *P* values were calculated with the Student *t*-test, *P < .05.

With respect to the brains, we first assessed changes in absolute numbers of CD3^+^, CD4^+^, and CD8^+^ T cells after Delta24-RGD therapy. There was a distinct time-dependent increase in intratumoral CD3^+^ T cells after therapy, reaching significance at 14 days (*P* < .05). This increase in TILs reflected a specific increase in CD8^+^ T cell numbers whereas the numbers of CD4^+^ T cells remained low (Figure 1A). By day 14, the ratio of CD8^+^ to CD4^+^ T cells was increased from 1.138 in PBS controls to 2.755 after Delta24-RGD therapy (*P* = .0835). Next, we assessed the differential expression of co-stimulatory and co-inhibitory molecules on brain-infiltrating CD3^+^ T cells (Figure 1B). Similar to splenocytes, low numbers of intratumoral 4-1BB^+^, OX40^+^, and CD40L^+^ T cells were detected in the brain. In contrast, there were high numbers of intratumoral CD28^+^ T cells after therapy and significantly more ICOS^+^ T cells at day 14 (*P* < .05). The number of co-inhibitory CTLA-4^+^, TIM-3^+^, and BTLA^+^ intratumoral T cells did not increase significantly after Delta24-RGD therapy. LAG-3^+^ TILs were significantly more abundant at 72 h and, importantly, PD-1^+^ T cells were significantly more abundant in the brains 14 days post-therapy (*P* < .05).

### High Density of Intratumoral PD-1^+^ T Cells After Delta24-RGD Therapy Inversely Correlates With Brain Tumor Size and Antitumor T-cell Activity *Ex Vivo*

**Fig. 2:**
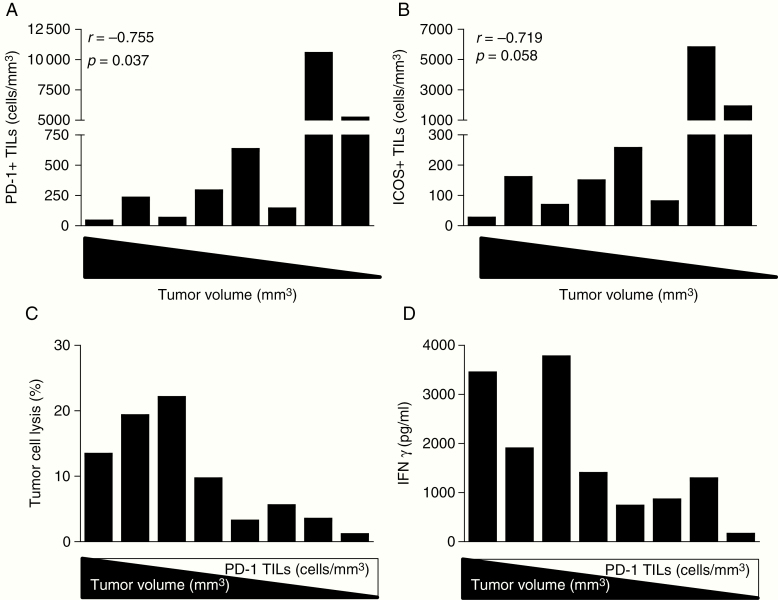
High density of intratumoral PD-1^+^ T cells after Delta24-RGD therapy inversely correlates with brain tumor size and ex vivo T cell activity. Expression of PD-1^+^ and ICOS^+^ TILs analyzed by flow cytometry are defined as numbers of positive CD3^+^ T cells per mm^3^ tumor volume and stratified by matched tumor volume (from large to small). (A) Large tumor volume was correlated with a low density of PD-1^+^ TILs (Spearman *r* = −0.755; *P* = .037). (B) The density of ICOS^+^ TILs was not significantly correlated with tumor volume (Spearman *r* = −0.719; *P* = .058). (C) Ex vivo cell cultures were established by dissociating brain tumors into single cells which were cultured in a live monitoring incubator for 5 days. The rate of tumor cell lysis was calculated using ImageJ software by comparing the images from day 0 to day 5 of the culture. Tumor cell lysis per TILs culture was correlated with the volume of the brain tumor from which the cultures were derived (Spearman *r* = 0.802; *P* = .022) and inversely correlated with the density of PD-1^+^ TILs (Spearman *r* = −0.762; *P* = .037). (D) IFNγ production in supernatant was significantly correlated with brain tumor volume (Spearman *r* = 0.802; *P* = .022) and inversely correlated with the density of PD-1^+^ TILs (Spearman *r* = −0.714; *P* = .058).

To understand the relevance of ICOS and PD-1 expression in brain TILs, we treated 8 glioma-bearing mice with Delta24-RGD and harvested the brains and spleens on day 14 after therapy. Brain tumor volumes ranged from 0 to 300 mm^3^ and were found to be inversely correlated to the density of intratumoral PD-1^+^ TILs (*P* < .05; Figure 2A), and, although not statistically significant, a trend toward correlation for ICOS^+^ TILs was observed (*P* = 0.058; Figure 2B). This indicates that the regression of brain tumors is accompanied by the presence of PD-1-expressing TILs. Brain tumor suspensions were plated at equal cell numbers and cultured in a live-cell imaging incubator. After 5 days, tumor cell lysis was calculated based on decreased confluency of tumor cells in the cell culture images and the supernatant was collected to assess IFNγ production by ELISA (Supplementary Figure S2). We observed that tumor cell lysis is positively correlated with IFNγ production, indicating that intratumoral T cells retain their capacity to perform cytotoxic effector functions ex vivo (<.01; Supplementary Figure S3). Interestingly, both tumor cell lysis and IFNγ production are correlated with tumor volume (*P* < .05), suggesting that tumors unresponsive to therapy—represented by the bigger tumors in this cohort—contain exhausted T cells that reactivate upon removal from their immune suppressive milieu. Both tumor cell lysis and IFNγ production are inversely correlated with the density of intratumoral PD-1^+^ T cells (*P* < .05) (Figure 2C and D). This counterintuitive observation may result from the PD-1-mediated negative feedback loop to limit the expansion of effector T cells during an immune response. We therefore hypothesize that regressed tumors contain a higher density of PD-1-expressing T cells that have experienced antigen and performed effector functions. Blockade of PD-1 on these T cells prolongs effector T-cell activity and expansion.

### Ex Vivo PD-1 Blockade Significantly Increases Tumor-Specific IFNγ Production

**Fig. 3:**
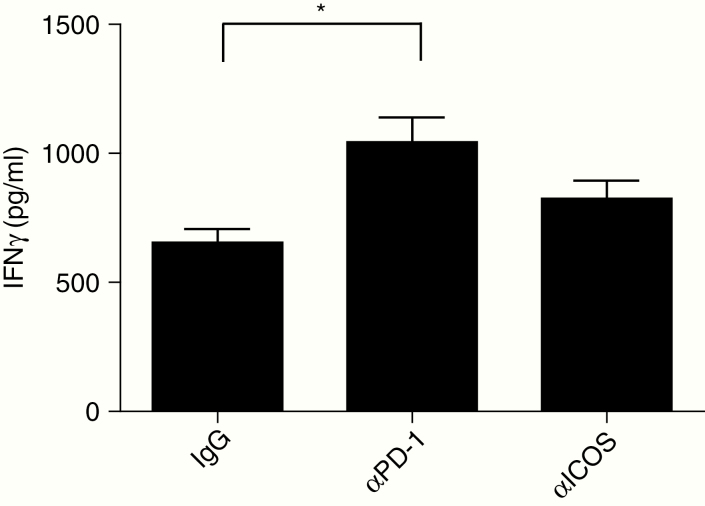
Splenocytes from glioma-bearing mice treated with Delta24-RGD produce significantly more IFNγ when cultured with PD-1 blockade. Splenocytes were harvested, processed into single cells, and co-cultured with GL261 cells with hamster IgG (IgG control), anti-PD-1 blocking antibody, or anti-ICOS agonist antibody. After 4 days, the supernatant was collected and IFNγ production was assessed using ELISA. Splenocytes from Delta24-RGD-treated mice produce IFNγ upon recognition of GL261 glioma cells (IgG bar). IFNγ production was significantly increased with anti-PD-1 blocking antibodies but not with ICOS agonist antibodies (*P* = .0029). *P*-value is calculated by ANOVA and corrected for multiple testing with the post-hoc Bonferroni test. *P < .05.

We have previously shown that splenocytes isolated from tumor-bearing mice treated with Delta24-RGD are able to recognize both tumor and virus antigens.^28^ To test the efficacy of antibody immunotherapy, splenocytes derived from glioma-bearing mice treated with Delta24-RGD were co-cultured with GL261 cells in the presence of anti-PD-1 (blocking) and anti-ICOS (agonist) antibodies. We observed that splenocytes derived from glioma-bearing mice treated with Delta24-RGD produced IFNγ upon recognition of the glioma antigens (Figure 3). The addition of a blocking anti-PD-1 antibody, and not of an agonist ICOS antibody, resulted in a significant increase in IFNγ production (*P* < .01).

### Low-Dose Delta24-RGD Combined With PD-1 Blockade Significantly Improves Survival in Syngeneic Mouse Models for Glioma

**Fig. 4:**
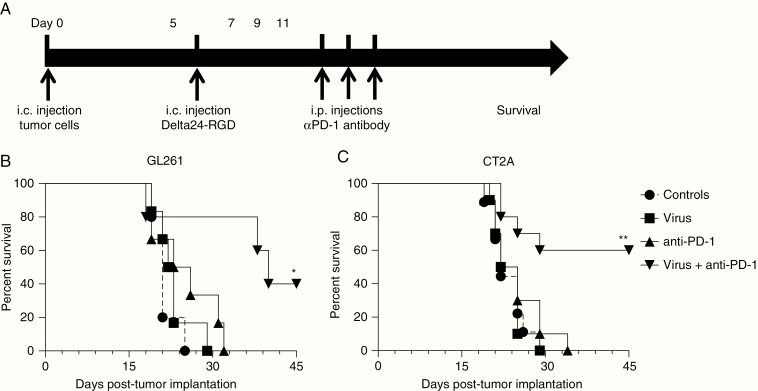
Low-dose Delta24-RGD combined with anti-PD-1 therapy significantly improves long-term survival. (A) Treatment schedule of a combination study with Delta24-RGD and anti-PD-1 antibody therapy. C57BL/6J mice were inoculated i.c. with 5 × 10^4^ GL261 or CT2A cells and after 5 days treated i.c. with 2 × 10^7^ pfu Delta24-RGD in 10 µL PBS and control mice were treated with 10 µL PBS. Anti-hamster PD-1 blocking antibodies (G4) or Hamster IgG (control) were injected intraperitoneally at doses of 200 µg/injection at days 7, 9, and 11 post-tumor implants. (B) Kaplan–Meier analysis showed a significant improvement of median survival to 40 days and 40% of mice surviving longer than 90 days (LTS) with combined Delta24-RGD therapy and anti-PD-1 therapy compared to all other groups in the orthotopic GL261 tumor model (*P* < .05). (C) Kaplan–Meier analysis showed improved overall survival (median survival undefined) and 60% LTS with combined Delta24-RGD and anti-PD-1 therapy in the orthotopic CT2A tumor model (*P* < .01). *P* values were calculated with the Log-Rank test. *P < .05, **P < .01.

To assess the combinatorial effects of viro-immunotherapy in vivo, a 10-fold lower dose of Delta24-RGD was chosen than the previously determined effective dose in the GL261 orthotopic mouse model,^28^ in order to determine the potential to synergize with anti-PD-1 antibody therapy (Figure 4A). The Delta24-RGD treatment or anti-PD-1 antibodies alone did not improve median survival compared to PBS-treated controls (*P* = .1654 and *P* = .3965 vs controls, respectively). However, mice treated with combined low-dose Delta24-RGD therapy and anti-PD-1 antibody therapy had a significantly increased median survival compared to either treatment modality alone (40 days vs 24.5 days for Delta24-RGD alone and 22.5 days for anti-PD-1 alone) and achieved 40% long-term survivors beyond 100 days versus none in the monotherapy groups (*P* < .05, Figure 4B).

The GL261 tumor model has been considered immunogenic due to the expression of MHC-I,^40^ we therefore also assessed the efficacy of the combined viro-immunotherapy in the non-immunogenic CT2A glioma model. Similar to the GL261 model, single therapy with low dose of Delta24-RGD or anti-PD-1 antibodies did not yield a survival benefit compared to controls (*P* = .9905 and *P* = .6349 vs controls, respectively) whereas combined Delta24-RGD and anti-PD-1 therapy significantly improved median survival (undefined vs 23.5 days for Delta24-RGD alone and 23.5 days for anti-PD-1 alone) and resulted in 60% long-term survivors (*P* < .01, Figure 4C). Together, these studies in 2 syngeneic mouse models support the hypothesis that the addition of anti-PD-1 to Delta24-RGD therapy for malignant glioma may improve therapeutic response rates.

Immunohistochemical staining of mouse brains obtained at sacrifice revealed the presence of scattered hexon-positive cells in the virus-treated tumors, with slightly more viral proteins detected in the Delta24-RGD than in the combination-treated group. Tumors from both Delta24-RGD-treated groups revealed higher numbers of CD4 and CD8 T cells compared to control tumors (Supplementary Figure S4).

### Delta24-RGD Combined With PD-1 Blockade Increases PD-1^+^ TILs

**Fig. 5:**
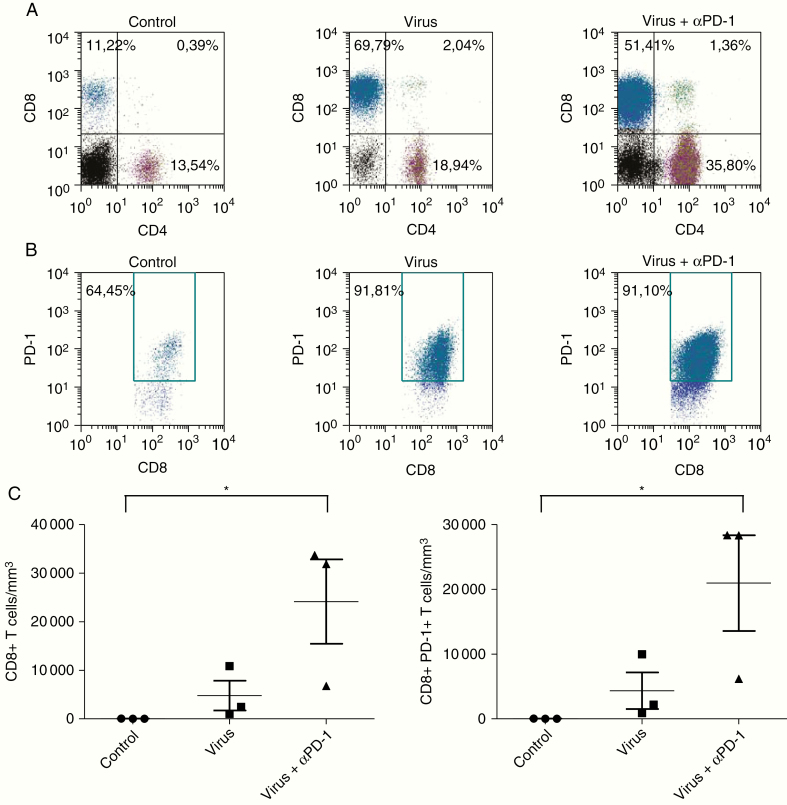
Low-dose Delta24-RGD combined with PD-1 blockade increases PD-1^+^ TILs. Mice bearing GL261 tumors (*n* = 3/group) and treated as described in Figure 4A were euthanized 14 days post-therapy and brains were processed into single cells for flow cytometry. (A) Flow cytometric analysis showed an increased percentage of CD8^+^ T cells (cells gated on CD3^+^ T cells) after Delta24-RGD therapy only (middle panel) and in combination with anti-PD-1 therapy (right panel). (B) Intratumoral CD8^+^ T cells highly express PD-1 (cells gated on CD3^+^CD8^+^ T cells). (C) Combination therapy significantly increased the absolute numbers of CD8^+^ T cells and PD-1^+^ CD8^+^ T cells per mm^3^ tumor. *P* values were calculated by ANOVA with post-hoc Tukey test. *P < .05.

To investigate whether combining Delta24-RGD with anti-PD-1 therapy affects the presence of PD-1 expression in gliomas, we isolated TILs from tumors obtained at day 14 post-therapy. Analysis by flow cytometry showed a marked increase in the percentage of CD8^+^ T cells upon Delta24-RGD therapy alone or combined with anti-PD-1 (Figure 5A). Interestingly, only combination therapy increased the percentage of CD4^+^ T cells confirming our previously published results that CD4^+^ T cells play a significant role in viro-immunotherapy.^29^ Both modalities increased the CD8/CD4 T-cell ratio, from 0.83 in PBS-treated controls to 3.68 after Delta24-RGD monotherapy and 1.44 after combination therapy and increased the percentage of PD-1^+^ CD8^+^ T cells from 64.45% to approximately 91%, respectively (Figure 5B). In terms of absolute numbers, we observed an increase in the density of CD8^+^ T cells after Delta24-RGD therapy. The addition of PD-1 blockade to Delta24-RGD therapy further increased the absolute numbers of intratumoral CD8^+^ T cells as well as PD-1^+^ CD8^+^ T cells (*P* < .05) (Figure 5C). We conclude that the absolute numbers of both CD8^+^ and PD-1 expressing CD8^+^ T cells are significantly increased upon the addition of anti-PD-1 therapy.

### Delta24-RGD Infection Induces Tumor-Dependent Changes in PD-L1 Expression in Low-Passage GBM Cell Cultures

As various anticancer treatments have been shown to induce upregulation of PD-L1 on the surface of tumor cells,^41^ we assessed whether Delta24-RGD infection also confers this effect. PD-L1 expression was measured on murine GL261 cells after infection with increasing doses of Delta24-RGD (MOI 10, 100, and 1000). Approximately 60% of untreated GL261 cells expressed PD-L1, and this percentage remained similar after infection with increasing MOIs of Delta24-RGD (*P* = .2318; Supplementary Figure S5).

**Fig. 6:**
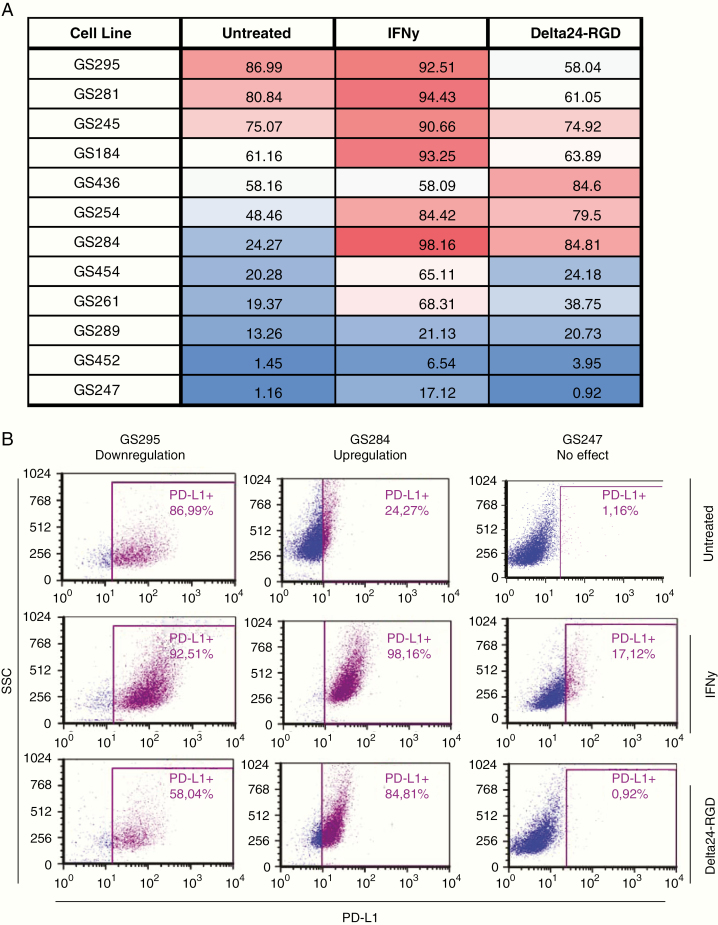
Delta24-RGD induces cell line specific PD-L1 expression in primary GBM cell lines. Low passage patient-derived cell cultures were cultured in the presence of 10 ng/mL IFNγ or with a cell culture-specific MOI Delta24-RGD dilution for 24 h. Cells were stained with 7-AAD and anti-human CD274 (PD-L1) and analyzed with flow cytometry. (A) A table reflecting PD-L1 expression in a panel of 12 primary GBM cell cultures at baseline and after IFNγ or Delta24-RGD treatment. (B) PD-L1 expression assessed with flow cytometry in 3 representative primary patient-derived GBM cell lines GS295 (left panel), GS284 (middle panel), and GS247 (right panel).

To gain insight into the effects of Delta24-RGD infection on PD-L1 expression in human GBM, patient-derived GBM cultures (*n* = 12) were infected with the IC_50_ dosage of Delta24-RGD or were treated with IFNγ, a known inducer of tumor PD-L1 expression.^15^ PD-L1 expression was assessed with flow cytometry after 24 h when cell viability was not yet affected by the virus (>85% 7-AAD negative tumor cells; data not shown). We observed extensive intertumoral variability in baseline expression of PD-L1, ranging from 1% to 87% positive cells (Figure 6A). Treatment with IFNγ significantly increased the expression of PD-L1 in every cell line (*P* = .0027) whereas treatment with Delta24-RGD yielded cell culture-specific responses in the regulation of PD-L1 expression (*P* = .2250; Supplementary Figure S6). For example, the cell line GS247 has low baseline PD-L1 expression that is not affected by Delta24-RGD, whereas GS284 has intermediate baseline PD-L1 expression that significantly increased after Delta24-RGD infection (Figure 6B). These results show that GBM cells express unique patterns of PD-L1 expression and that Delta24-RGD therapy induces tumor-specific alterations in PD-L1 expression.

## Discussion

Sub-optimal response rates to checkpoint inhibitors have spurred the quest to identify approaches to sensitize the tumor microenvironment prior to immunotherapy. OVs are promising candidates for this task and a growing number of studies support their use as immune-priming agents to turn non-immunogenic (“cold”) tumors into immunogenic (“hot”) tumors. We previously showed that Delta24-RGD affects local innate immune cells in GBM by inducing phenotypic skewing of pro-tumor M2-like macrophages toward an antitumor M1-like phenotype, therewith inducing a pro-inflammatory environment.^42^ Various OVs based on adenovirus, HSV, reovirus, VSV, and measles virus are currently under investigation in combination with immune checkpoint inhibitors.^31,43–46^ The effect of the oncolytic adenovirus Delta24-RGD on the expression of immune checkpoints within the local GBM milieu has thus far not been described. In this study, we assessed the kinetic changes in expression of 10 co-signaling molecules on intratumoral T cells after Delta24-RGD therapy with the aim to systematically select a candidate for combination therapy. Our study is the first to show that a low dose of Delta24-RGD is already sufficient to alter the tumor microenvironment and prime the immune system for effective synergy with anti-PD-1 antibody therapy.

OVs have emerged as potent immune-activating therapeutic agents. This development translated into a shift within the OV field from improving cytolytic capacities of the viruses into enhancing their immunogenicity.^47^ Our laboratory previously showed that the interplay between the immune system and Delta24-RGD is crucial in the propagation of an antitumor immune response.^28,29^ Both antiviral and antitumor T-cell responses are induced, suggesting that the adaptive immune system balances between virus and tumor clearance. To tip the scales in favor of antitumor immune response, additional T-cell activation would aid infiltrating immune cells in combating the highly suppressive GBM. In immune-competent mouse models for GBM, effective strategies have been described involving various OVs combined with various immune checkpoint antibodies including anti-PD-1 and OX40.^31,43–46^ In GL261 tumors, in particular, vesicular stomatitis virus and anti-PD-1 therapy significantly increased IFNγ production through an antitumor and antiviral response, suggesting that PD-1 blockade resulted in re-activation of antitumor Th1 T-cell responses.^45^ To build upon our previously established results we tested our hypothesis in immune-competent C57BL/6 mice with orthotopic GL261 tumors as well as in the less immunogenic CT2A model. Both experiments yielded similar results showing that Delta24-RGD effectively primes the tumor microenvironment and sensitizes to anti-PD-1 therapy, regardless of the brain tumor cell type. A recent study shows that Delta24-RGD therapy expressing the immunostimulatory OX40 ligand resulted in a cure of subcutaneous melanoma tumors and distant untreated tumors, including intracranial tumors, demonstrating that OVs can be successfully armed with co-signaling molecules to boost systemic antitumor immune responses.^48^

Our results confirm the antigen-specific antitumor response shown by increased IFNγ production in co-cultures with virus-treated splenocytes and GL261 cells that can be further enhanced with anti-PD-1 antibodies. The local tumor microenvironment was significantly impacted by the single low-dose delivery of Delta24-RGD. We observed an increase in CD8^+^ TILs expressing mostly ICOS and PD-1. Furthermore, brain tumor size was inversely correlated with PD-1^+^ T-cell densities in the tumor, suggesting that active tumor regression is correlated with an increased local presence of PD-1^+^ T cells. Interestingly, we showed that active tumor cell lysis and IFNγ production were only detected in ex vivo cultures derived from larger tumors containing low PD-1^+^ TILs densities confirming the potential of these TILs to produce IFNγ when adaptive immune resistance has not yet occurred. We thus hypothesized that PD-1^+^ TILs constitute anergic antigen-specific T cells ready for re-activation upon sequential PD-1 blockade. Indeed, combination therapy with Delta24-RGD and anti-PD-1 significantly improved overall survival and increased the density of intratumoral PD-1^+^ CD8^+^ T cells.

Another relevant molecule in anti-PD-1 therapy, and a potential biomarker for response to therapy, is PD-L1. The expression of PD-L1 is normally increased in an inflammatory microenvironment to down-regulate ongoing immune activation, but this mechanism is hijacked by tumor cells to specifically inhibit T-cell effector functions.^49^ Taube et al.^50^ showed that PD-L1^+^ melanomas correlated with increased PD-1^+^ expression in TILs and were generally associated with improved long-term survival with anti-PD-1 therapy. PD-L1 expression in GBM was previously reported to be highly variable between patients.^51^ This observation was confirmed in our panel of low-passage patient-derived GBM cultures, with PD-L1 expression varying from 1% to 87% positive cells. Infection with Delta24-RGD induced diverging effects on PD-L1 expression ranging from downregulation to upregulation of PD-1 levels. This indicates that Delta24-RGD therapy induces tumor-specific changes in PD-L1 expression that could impact sequential treatment with anti-PD-1 therapy and may argue for an individualized therapeutic approach.

In conclusion, low-dose Delta24-RGD therapy effectively sensitizes murine GL261 and CT2A gliomas to sequential anti-PD-1 therapy. This combined viro-immunotherapeutic approach synergizes to overcome adaptive immune resistance induced by intratumoral PD-1 expressing CD8^+^ T cells, leading to an effective IFNγ-mediated antitumor immune response and long-term cure of glioma-bearing mice. The selective infection and killing of GBM cells and subsequent immune-modulatory capabilities qualify Delta24-RGD therapy as an attractive candidate for combination with immune checkpoint inhibitors. The combination of Delta24-RGD and PD-1 blockade is currently under investigation in a clinical phase II study for recurrent GBM (NCT02798406).

## Funding

This work was supported by the foundation STOPbraintumors.org and the Erasmus University Medical Center Mrace program.

## Supplementary Material

vdaa011_suppl_Supplementary_Figure_S1

vdaa011_suppl_Supplementary_Figure_S2

vdaa011_suppl_Supplementary_Figure_S3

vdaa011_suppl_Supplementary_Figure_S4

vdaa011_suppl_Supplementary_Figure_S5

vdaa011_suppl_Supplementary_Figure_S6

vdaa011_suppl_Supplementary_Table_Figure_Legends

## Notes

**Authorship Statement**: Experimental design: Z.B., C.B., E.V.B., J.C., E.S., T.P., J.K., R.D., and M.L.M.L. Implementation: Z.B., C.B., E.V.B., J.C., E.S., T.P., J.K., D.R., M.V.D.K., A.V.D.P., and D.M. Analysis and interpretation of data: Z.B., C.B., E.V.B., J.C., J.K., R.D., and M.L.M.L. Writing and revision manuscript: Z.B., C.B., J.C., E.V.B., J.K., M.V.D.K., A.V.D.P., D.M., S.S., C.D., M.L., R.D., and M.L.M.L. All authors have read and approved the final version of this manuscript.
