# Supplementary figures and images for: Low-dose oncolytic adenovirus therapy overcomes tumor-induced immune suppression and sensitizes intracranial gliomas to anti-PD-1 therapy
Source: Neurooncol Adv. 2020 Feb 3;2(1):vdaa011. doi: 10.1093/noajnl/vdaa011 (PMC7212906; doi:10.1093/noajnl/vdaa011)

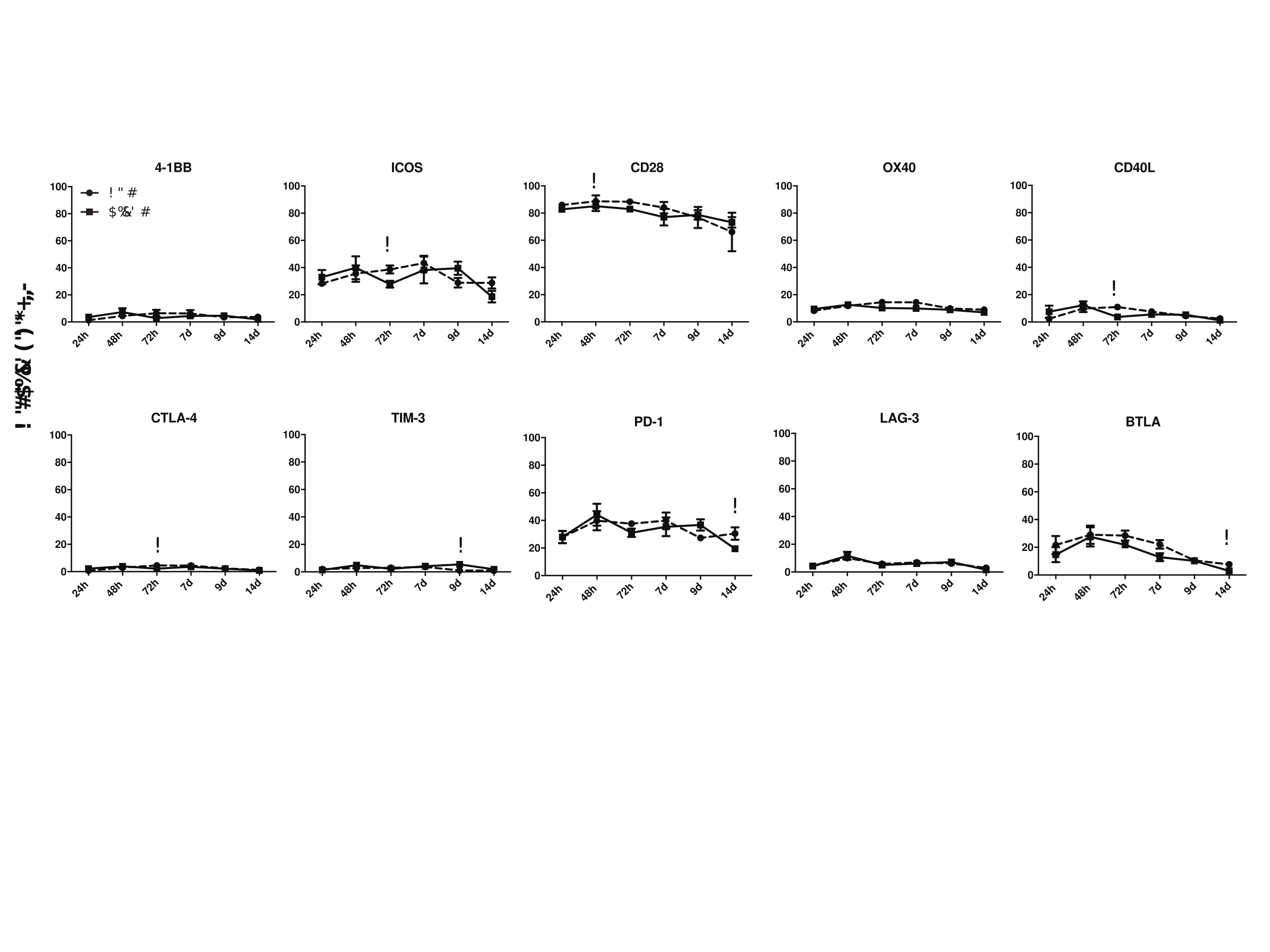

Supplement: vdaa011_suppl_Supplementary_Figure_S1 [file vdaa011_suppl_supplementary_figure_s1.png]

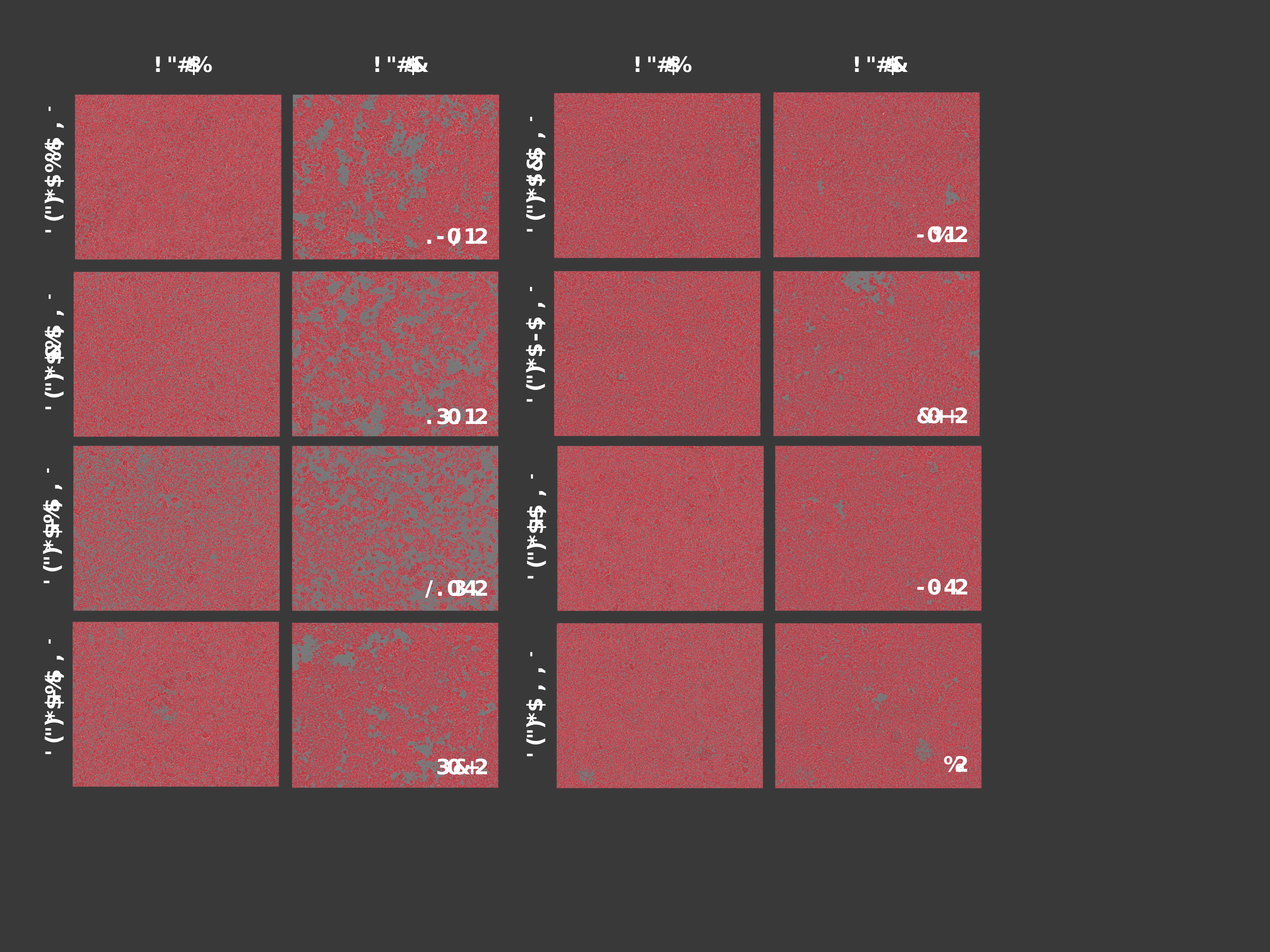

Supplement: vdaa011_suppl_Supplementary_Figure_S2 [file vdaa011_suppl_supplementary_figure_s2.png]

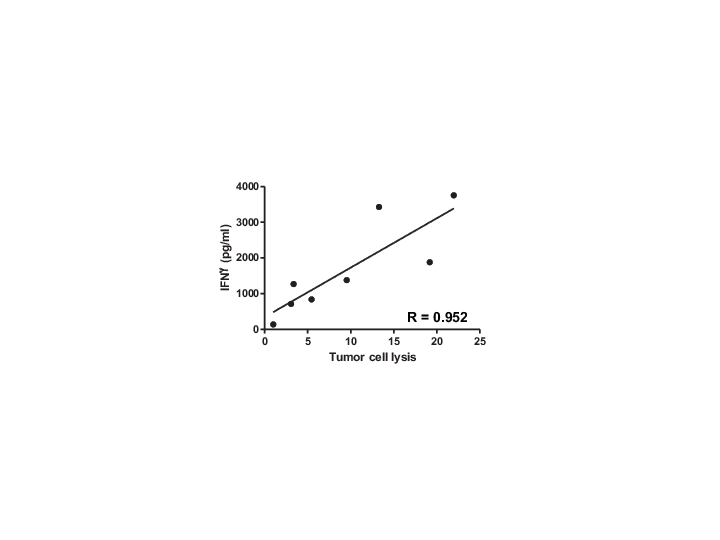

Supplement: vdaa011_suppl_Supplementary_Figure_S3 [file vdaa011_suppl_supplementary_figure_s3.png]

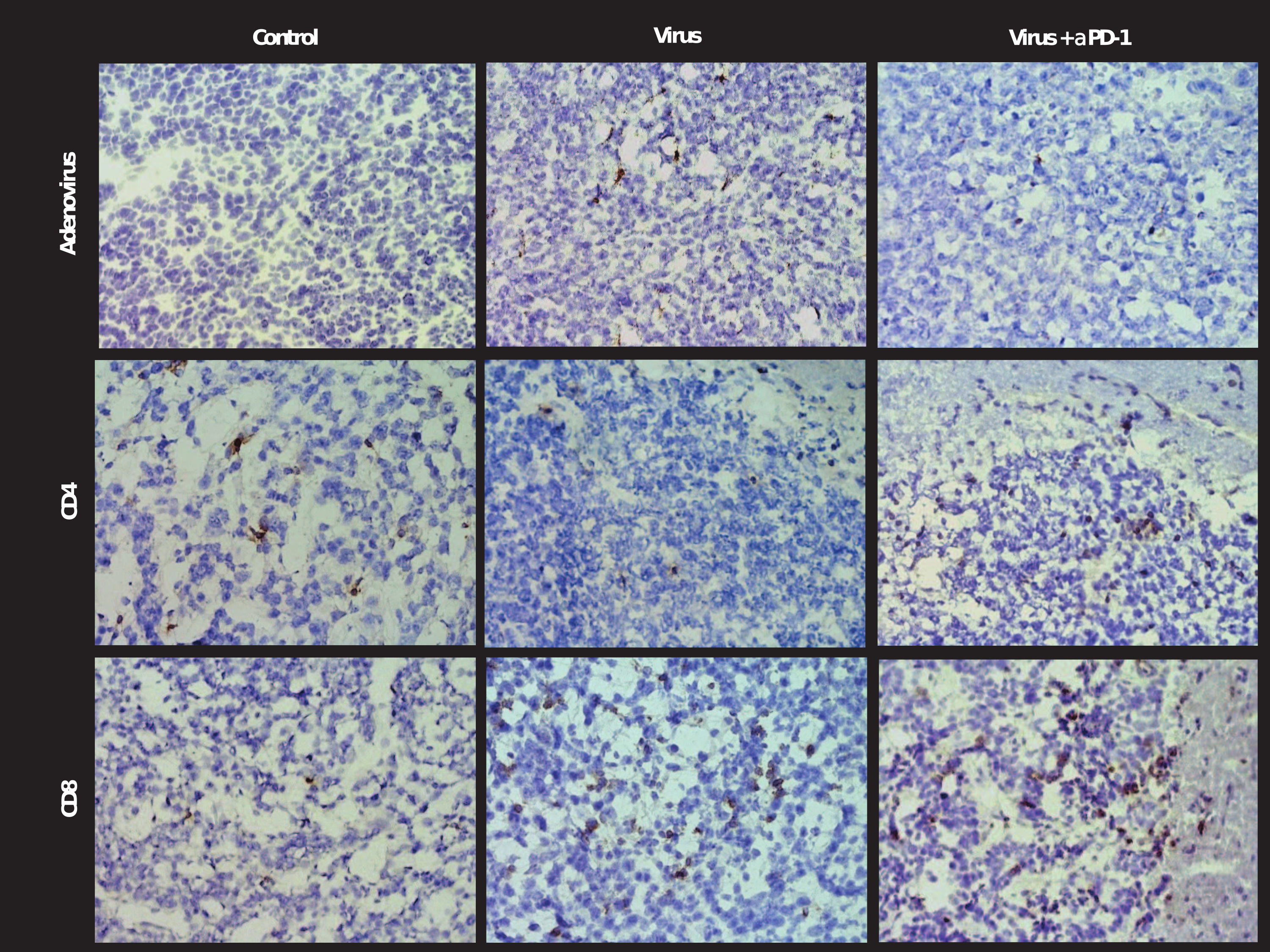

Supplement: vdaa011_suppl_Supplementary_Figure_S4 [file vdaa011_suppl_supplementary_figure_s4.png]

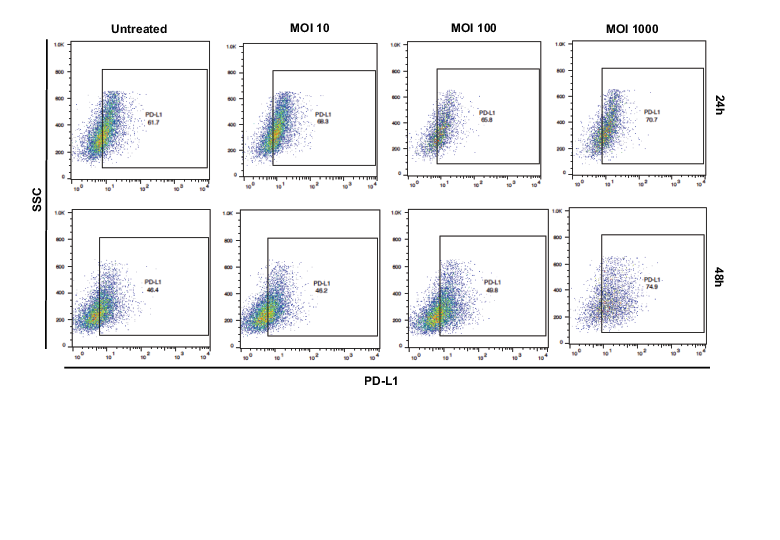

Supplement: vdaa011_suppl_Supplementary_Figure_S5 [file vdaa011_suppl_supplementary_figure_s5.png]

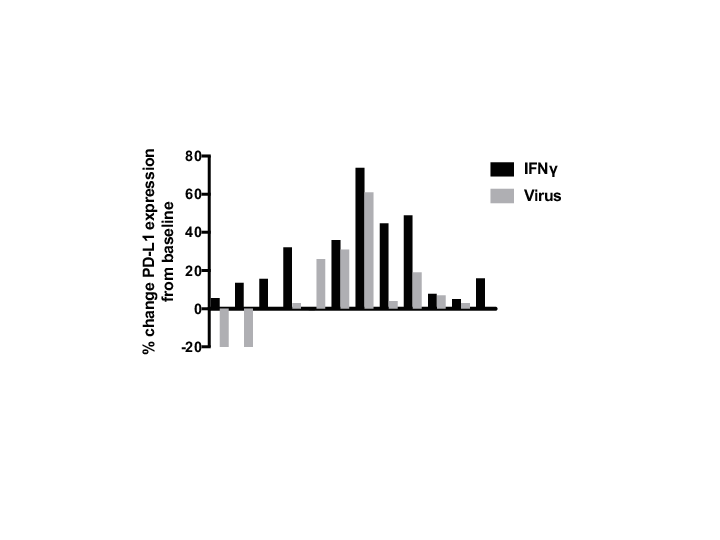

Supplement: vdaa011_suppl_Supplementary_Figure_S6 [file vdaa011_suppl_supplementary_figure_s6.png]
