# Supplementary material for: Low-dose oncolytic adenovirus therapy overcomes tumor-induced immune suppression and sensitizes intracranial gliomas to anti-PD-1 therapy
Source: Neurooncol Adv. 2020 Feb 3;2(1):vdaa011. doi: 10.1093/noajnl/vdaa011 (PMC7212906; doi:10.1093/noajnl/vdaa011)
Supplement: vdaa011_suppl_Supplementary_Table_Figure_Legends [file vdaa011_suppl_supplementary_table_figure_legends.docx]

**SUPPLEMENTARY TABLE AND FIGURE LEGENDS**

**Supplementary Table 1. Anti-mouse antibodies for flow cytometry:**

| **Antibody** | **Clone** | **Fluorophore** | **Company** |
| --- | --- | --- | --- |
| CD3 | 145-2C11 | PerCP | eBioscience |
| CD4 | GK1.5 | PE-Cy7 | eBioscience |
| CD8α | 53-6.7 | APC-Cy7 | eBioscience |
| CD278 (ICOS) | 7E.17G9 | FITC | eBioscience |
| CD28 | 37.51 | PE | eBioscience |
| CD154 (CD40L) | MR1 | PE | eBioscience |
| CD223 (LAG-3) | C9B7W | APC | eBioscience |
| CD279 (PD-1) | RMP1-30 | APC | eBioscience |
| CD134 (OX-40) | OX-86 | PE | Biolegend |
| CD152 (CTLA-4) | UC10-4B9 | PE | Biolegend |
| CD272 (BTLA) | 8F4 | APC | Biolegend |
| TIM-3 | RMT3-23 | APC | Biolegend |
| CD137 (4-1BB) | 1AH2 | FITC | BD Biosciences |

**Supplementary Figure S1. Delta24-RGD therapy does not yield a differential expression of T cell co-signaling molecules in splenocytes.** C57BL/6J mice bearing i.c. GL261 gliomas were treated on day 5 by i.t. injection of 2x 10^7^ pfu Delta24-RGD in 10 ul PBS or 10ul PBS. Mice were sacrificed after 24h, 48h, 72h, 7 days, 9 days and 14 days post-therapy and spleens were processed for flow cytometry (n= 3-5 mice per time-point per group). Expression of ten co-signaling molecules was assessed in CD3^+^ T cells isolated from splenocytes of mice treated with Delta24-RGD and compared to controls. P values were calculated with the student t-test. *, P < 0.05.

**Supplementary Figure S2. Live cell monitoring of *ex vivo* cell cultures derived from intracranial GL261 gliomas treated with Delta24-RGD reveal active tumor cell lysis.** C57BL/6J mice bearing GL261 tumors were treated on day 5 with 2x 10^7^ pfu Delta24-RGD in 10 ul PBS and were sacrificed after 14 days post-therapy (n= 8 mice). Ex vivo cell cultures were established by dissociating brain tumors into single cells. These cell suspensions contain both GL261-derived tumor cells and immune cells and were cultured in a live cell imaging incubator. Images reflect start and end (day 5) of *ex vivo* live monitoring and were analyzed with ImageJ software to calculate the rate of tumor cell lysis based on loss of tumor cell density. The density of red cells on day 0 was normalized to 100% and relative loss of red tumor cells compared to the gray area on day 5 was expressed in percentage loss compared to day 0. The images are ranked top to bottom in descending order of brain tumor size (big to small) from which the cultures originate.

**Supplementary Figure S3. Tumor cell lysis is positively correlated with IFNγ** **production in *ex vivo* cultures.** Brain suspensions containing tumor cells and immune cells from mice treated with Delta24-RGD were cultured for 5 days in a live cell imaging incubator. Supernatant was collected at the end of culture and IFNγ was assessed using ELISA. Tumor cell lysis was calculated as described in Fig. S2. There was a significant positive correlation between tumor cell lysis and IFNγ production (Spearman R = 0.9524; p < 0.01). Correlation was calculated with the Spearman’s rank correlation coefficient.

**Supplementary Figure S4. Delta24-RGD therapy effectively infects intracranial brain tumors and increases the influx of CD4 and CD8 T cells.** Brains were collected from untreated, Delta24-RGD treated and combination therapy treated mice at time of sacrifice. Sections of 10 µm from snap frozen brains were stained with antibodies against adenovirus, CD4 or CD8 using DAB chromogen. Adenovirus was present in Delta24-RGD and combination treated brain tumors indicating an active delivery and persistence of the virus. As confirmed with flow cytometry (Figs. 1 and 5), CD4 T cells were moderately increased after virus therapy whereas a marked increase in CD8 T cells was observed after both Delta24-RGD therapy alone and in combination with anti-PD-1 therapy.

**Supplementary Figure S5. Infection with Delta24-RGD does not yield changes in PD-L1 expression in murine GL261 tumor cells.** Murine GL261 cells were infected with increasing MOIs (10, 100, and 1000) of Delta24-RGD and PD-L1 expression was assessed with flow cytometry after 24h (top row) and 48h (bottom row) of infection. Infection with Delta24-RGD does not induce changes in PD-L1 expression in GL261 cells compared to untreated cells (p = 0.2318). P values were calculated with ANOVA.

**Supplementary Figure S6. Infection with Delta24-RGD yields cell-specific changes in PD-L1 expression in human GBM cell lines.** Low passage serum-free cell cultures established from patient GBM tumors were cultured in the presence of 10 ng/mL IFNγ or with a cell culture-specific MOI Delta24-RGD dilution for 24h. Cells were stained with 7-AAD and anti-human PD-L1 and analyzed with flow cytometry. Human GBM cell lines treated with IFNγ significantly increased PD-L1 expression compared to baseline (p = 0.0027). Delta24-RGD infection induced cell-line specific changes in PD-L1 expression compared to baseline (p = 0.2250). The expression of PD-L1 within each cell line is depicted after IFNγ treatment (black bar) or after Delta24-RGD therapy (gray bar). P values were calculated with the paired student t-test.
